# Genotyping Canadian *Cyclospora cayetanensis* Isolates to Supplement Cyclosporiasis Outbreak Investigations

**DOI:** 10.3390/microorganisms10020447

**Published:** 2022-02-15

**Authors:** Christine A. Yanta, John R. Barta, Antoine Corbeil, Hervé Menan, Karine Thivierge, Robert Needle, Muhammad Morshed, Brent R. Dixon, James D. Wasmuth, Rebecca A. Guy

**Affiliations:** 1 National Microbiology Laboratory at Guelph, Division of Enteric Diseases, Public Health Agency of Canada, Guelph, ON N1G 3W4, Canada; christine.yanta@phac-aspc.gc.ca; 2 Department of Pathobiology, Ontario Veterinary College, University of Guelph, Guelph, ON N1G 2W1, Canada; jbarta@ovc.uoguelph.ca; 3 Public Health Ontario, Toronto, ON M5G 1M1, Canada; antoine.corbeil@oahpp.ca; 4 Québec Public Health Laboratory, Ste-Anne-de-Bellevue, QC H9X 3R5, Canada; rvmenan@yahoo.fr (H.M.); karine.thivierge@inspq.qc.ca (K.T.); 5 Public Health and Microbiology Laboratory, Eastern Health, St John’s, NL A1A 3Z9, Canada; robert.needle@easternhealth.ca; 6 BC Centre for Disease Control Public Health Laboratory, Vancouver, BC V5Z 4R4, Canada; muhammad.morshed@bccdc.ca; 7 Bureau of Microbial Hazards, Food Directorate, Health Canada, Ottawa, ON K1A 0K9, Canada; brent.dixon@hc-sc.gc.ca; 8 Department of Ecosystem and Public Health, Faculty of Veterinary Medicine, University of Calgary, Calgary, AB T2N 4Z6, Canada; jwasmuth@ucalgary.ca

**Keywords:** *Cyclospora cayetanensis*, cyclosporiasis, genotyping, targeted amplicon deep sequencing (TADS), Canadian outbreaks, foodborne pathogen

## Abstract

*Cyclospora cayetanensis* is an emerging foodborne parasite that causes cyclosporiasis, an enteric disease of humans. Domestically acquired outbreaks have been reported in Canada every spring or summer since 2013. To date, investigations into the potential sources of infection have relied solely on epidemiological data. To supplement the epidemiological data with genetic information, we genotyped 169 Canadian cyclosporiasis cases from stool specimens collected from 2010 to 2021 using an existing eight-marker targeted amplicon deep (TADS) scheme specific to *C. cayetanensis* as previously described by the US Centers for Disease Control and Prevention (CDC). This is the first study to genotype Canadian *Cyclospora cayetanensis* isolates, and it focuses on evaluating the genotyping performance and genetic clustering. Genotyping information was successfully collected with at least part of one of the markers in the TADS assay for 97.9% of specimens, and 81.1% of cyclosporiasis cases met the minimum requirements to genetically cluster into 20 groups. The performance of the scheme suggests that examining cyclosporiasis cases genetically will be a valuable tool for supplementing epidemiological outbreak investigations and to minimize further infections. Further research is required to expand the number of discriminatory markers to improve genetic clustering.

## 1. Introduction

*Cyclospora cayetanensis* is the causative agent for the foodborne illness cyclosporiasis. This human gastrointestinal disease displays symptoms similar to other enteric pathogens, including watery diarrhea, abdominal cramping, and weight loss [1]. In Canada, the first cyclosporiasis outbreak was reported in 1996 [2] prior to becoming a nationally notifiable disease in 2000. Multijurisdictional foodborne outbreaks of locally acquired cases have been declared yearly during the spring or summer months since 2013 [3]. Previous Canadian foodborne cyclosporiasis outbreaks have been associated with a variety of food vehicles imported from endemic regions, including fresh berries (e.g., raspberries, blackberries) [3,4,5,6], fresh herbs (e.g., basil, cilantro) [7,8], leafy greens [9], and other fresh produce (e.g., sugar snap peas) [10].

Currently, Canadian outbreak investigations are conducted using epidemiological case-linkage data to identify initial source(s) of infection because no laboratory subtyping method has been validated. However, the long incubation period of *C. cayetanensis* (2–14 days), and the delay between the onset of symptoms and the laboratory results, makes trace-back analysis challenging. Due to its short shelf life, fresh produce epidemiologically linked to outbreaks is typically no longer available for testing once the case is confirmed in the laboratory. In addition, the common food vehicles associated with *Cyclospora* infections are often mixed with other fresh produce when consumed. These epidemiologic challenges combined with the lack of genetic information for the pathogen make it difficult to identify the parasite-contaminated food item common to the outbreak. Consequently, either outbreak investigations are resolved with significant uncertainty, or the source of infection remains unidentified altogether [11].

With decreased sequencing costs and increased computational power, whole genome sequencing (WGS) strategies are being used more routinely to identify and genetically characterize microbes, especially bacteria [12]. However, WGS methods for routine genotyping of *C. cayetanensis* infections are impractical because no propagation method currently exists. The parasite must be obtained directly from the clinical stool specimen and purified using discontinuous density gradient centrifugations with optional flow cytometry [13]. The isolation of adequate amounts of genomic DNA presents another challenge because standard diagnostic fecal samples contain only picogram amounts of *C. cayetanensis* DNA [14] that is insufficient for WGS library preparation. Finally, the whole genome of *C. cayetanensis* is relatively large (~44.4 Mbp), and there is currently no complete, closed reference genome available. As a result, a multilocus sequence typing (MLST) scheme is the best approach for routinely genotyping cyclosporiasis cases.

There have been several studies evaluating genetic markers to subtype *C. cayetanensis* infections [15,16,17,18,19,20,21]. Guo et al. [15] developed the MLST, which consisted of five microsatellite nuclear markers, but difficulties in PCR amplification and heterozygosity in the sequencing chromatograms were encountered [15,16]. With the improved availability of draft genomes, alternative markers were identified in either the nuclear or the mitochondrial genomes [18,19,20,21]. Of these, Nascimento et al. [21] described a new targeted amplicon deep sequencing (TADS) MLST approach to genotype *C. cayetanensis* infections. This scheme includes six nuclear (Nu_CDS1, Nu_CDS2, Nu_CDS3, Nu_CDS4, Nu_378, Nu_360i2) and two mitochondrial (Mt_MSR, Mt_Cmt) markers. The US Centers for Disease Control and Prevention (CDC) has described an ensemble-based method to cluster specimens together, which is designed to account for the sexual recombination of the *C. cayetanensis* genome that occurs [19,21,22]. This method was shown to have a 90–94% sensitivity and 99% specificity in clustering 2019 outbreak specimens genetically compared with epidemiologically defined cyclosporiasis clusters [21,22], demonstrating the potential for genetic clustering in the support of outbreak investigations.

In this study, we apply the targeted amplicon deep sequencing (TADS) assay originally described by Nascimento et al. [21] and Barratt et al. [22] to stool specimens from Canadian cyclosporiasis cases that were collected from 2010 to 2021 to further the molecular understanding of *C. cayetanensis* infections occurring in Canadians. This is the first study to apply molecular techniques on Canadian *C. cayetanensis* isolates. Data analysis was performed using the CDC’s improved genotyping system [22]. We describe the genotypes found within these Canadian samples and evaluate the genetic clusters generated to determine whether this tool shows potential in complementing epidemiological analyses to aid in outbreak mitigation.

## 2. Materials and Methods

### 2.1. Clinical Stool Specimens

A total of 187 human stool specimens positive for *C. cayetanensis* were received at the National Microbiology Laboratory (NML) at Guelph from four provincial public health partners: Public Health Ontario (PHO) Laboratory, Laboratoire de Santé Publique du Québec (LSPQ), British Columbia Centre for Disease Control (BCCDC) Public Health Laboratory, and Newfoundland Public Health Laboratory (NPHL). These specimens represent 169 cyclosporiasis case patients and were verified as *C. cayetanensis* positive at their respective provincial health laboratories from 2010 to 2021 prior to being shipped to NML. The samples were either received frozen without preservatives or on ice when suspended in the transport medium Cary-Blair, and were stored at −20 and 4 °C, respectively, prior to processing. Specimens from 2010 to 2019 were processed in 2019 and 2020, and specimens from 2020 and 2021 were processed in their respective years.

### 2.2. DNA Extraction

The stool samples were mixed and aliquoted into 0.2 g portions prior to being processed with a QIAamp Fast DNA Stool Mini Kit (Qiagen, Hilden, Germany) following the manufacturer’s instructions with modifications. Prior to adding Buffer AL to the supernatant, oocysts were pelleted by centrifuging the sample at 5000× *g* for 5 min. Half of the supernatant was removed into a new microcentrifuge tube, and the resulting pellet was subjected to 1 min of bead beating with Lysing Matrix Y (MP Biomedicals, Solon, OH, USA) at 6 m/s on Omni Bead Ruptor 24 (Omni International, Kennesaw, GA, USA). The supernatant was collected into a new microcentrifuge tube, and the beads were washed using the supernatant collected prior to bead beating. To ensure no beads were carried over, the tube was briefly centrifuged, and the supernatant was collected. At the end of the manufacturer’s protocol, DNA was eluted in two rounds of 50 µL of Buffer ATE and stored at −20 °C until use.

### 2.3. PCR Amplification and Cleanup

Eight PCRs were conducted on all specimens, representing six nuclear markers (CDS1, CDS2, CDS3, CDS4, HC378, and HC360i2) and two mitochondrial markers (Mt-Junction, MSR) as described by Nascimento et al. [21] and Barratt et al. [22], with minor modifications. For the nuclear gene targets, a KAPA HiFi HotStart PCR Kit (Roche, Basel, Switzerland) was used, whereas the Invitrogen Platinum Taq (Invitrogen by Life Technologies, Carlsbad, CA, USA) polymerase was used for the mitochondrial gene targets. The modified PCR reaction conditions are outlined in Table 1. For all eight gene markers, gBlock^TM^ gene fragments were designed to act as the positive control. The primers and gBlock^TM^ gene fragments were synthesized by Integrated DNA Technologies (IDT, Coralville, IA, USA). Finally, bovine serum albumin (BSA) heat shock fraction (Sigma-Aldrich, St. Louis, MO, USA) was added to each reaction at a final concentration of 300 ng/µL to reduce inhibition.

Before 2020, amplicons were visualized on a FlashGel DNA Cassette (Lonza Group, Basel, Switzerland). For specimens received in 2020 and 2021, PCR fragments were visualized using a QIAxcel DNA Screening cartridge (Qiagen, Hilden, Germany). Samples with positive bands were purified and normalized using a SequalPrep^TM^ Normalization Plate (96) Kit (Applied Biosystems by Thermo Fisher Scientific, Foster City, CA, USA) following the manufacturer’s protocols. For 2020 and 2021 specimens, amplicons were purified using AMPure XP beads (Beckman Coulter, Brea, CA, USA) and quantified using a NanoDrop^TM^ 8000 Spectrophotometer (Thermo Fisher Scientific, Waltham, MA, USA) for a more consistent normalization process. Finally, all eight clean and normalized amplicons were pooled for each sample for DNA sequencing preparation.

### 2.4. DNA Sequencing

A sequencing library of all the combined amplicons was prepared using a Nextera XT DNA Library Preparation Kit (Illumina, San Diego, CA, USA) following modifications as outlined in Nascimento et al. [21]. The library fragment size was measured using the Agilent 2100 Bioanalyzer system (Agilent Technologies, Santa Clara, CA, USA), and the concentration for each sample was measured using a Qubit^TM^ Fluorometer using a Qubit^TM^ dsDNA High Sensitivity Assay Kit (Invitrogen by Life Technologies, Carlsbad, CA, USA). Each sample was diluted to a final concentration of 4 nM prior to being further diluted to 10–15 pM (dependent on fragment size) for loading onto the cartridge. The libraries were then sequenced on an Illumina MiSeq platform using an MiSeq Reagent Nano Kit v2 (500 cycles, 2 × 250 bp) (Illumina, San Diego, CA, USA).

### 2.5. Sequence Analysis

The haplotypes for the eight markers for each specimen were determined using a Haplotype Caller module (Module 1) of the CDC’s Complete *Cyclospora* typing workflow (alpha test) on GitHub that captures novel haplotypes [19,21,22]. This module splits all markers, except for Mt_Cmt, into ~100 bp subsequent segments to alleviate haplotype misclassification from PCR-induced chimeras in the data [22]. As a result, the four Nu_CDS markers were split into two parts (Part A and Part B), the Nu_378 was split into four parts (Part A through D), and both the Nu_360i2 and Mt_MSR markers were split into six parts (Part A through F). For further information about splitting the markers into parts, refer to the Supplemental File S1 and the Supplementary Materials of Barratt et al. [22].

### 2.6. Genetic Clustering

Genetic distances were computed for downstream genetic clustering using the Calculation of a Distance Matrix module (Module 2) of the CDC’s Complete *Cyclospora* typing workflow (alpha test) on GitHub [19,21,22] using default parameters. This module uses the Eukaryotyping tool that was designed to compute genetic distances for specimens with missing data and heterozygosity at select markers using an ensemble-based method comprising two algorithms: one heuristic algorithm and a second Bayesian algorithm [19]. In order for an isolate to be clustered, genotyping data must be present for at least any five markers, or at least four markers if three from the following list were successfully sequenced: Nu_378, Nu_360i2_Mt_Cmt, and Mt_MSR [21]. The pairwise matrix generated from the Eukaryotyping tool was clustered and visualized following Nascimento et al. [21] in R (v4.1.4) using both ‘cluster’ (v2.1.0) and ‘ggtree’ (v2.0.0) packages. The number of genetic clusters was determined using the Genetic Cluster Delineation module (Module 3) of the typing workflow [19,21,22].

### 2.7. CDC Proficiency Panels

Proficiency panels comprising sets of clinical fecal specimens containing *C. cayetanensis* parasites of a known genotype set out by the CDC were genotyped in our lab in a blinded manner at the beginning of both the 2020 and 2021 outbreak seasons using the methods described above. Our lab met the CDC’s standards for both panels by observing a correct genotype from each specimen.

## 3. Results

### 3.1. Clinical Specimen Overview

**Figure 1:**
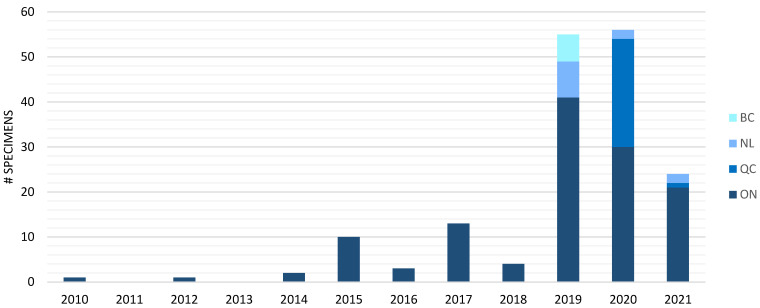
Yearly and provincial distribution of the 169 *Cyclospora cayetanensis*-positive unpreserved fecal specimens from cyclosporiasis case patients received at the NML in Guelph from 2010 to 2021 from four provinces: Ontario (ON), Québec (QC), Newfoundland and Labrador (NL), and British Columbia (BC).

In total, 187 unpreserved *C. cayetanensis* specimens from 169 case patients were collected from 2010 to 2021. The distribution of unpreserved specimens from case patients received at NML for analyses compared with the total laboratory-confirmed cases for the years 2014 to 2021 is as follows: 2% (2/85), 8% (10/131), 3% (3/87), 8% (13/158), 3% (4/123), 19% (55/296), 14% (56/399), and 32% (24/75). Overall, these represent 12% (167/1354) of laboratory confirmed cases in Canada from 2014 to 2021. This study also included one sample from 2010 and one sample from 2012, but the total number of specimens for those years is unknown. No samples were received in 2011 and 2013. The yearly distribution of unpreserved specimens from the 169 cyclosporiasis case patients received from the four provincial laboratories is outlined in Figure 1. The majority of specimens (74.6%) were received from Public Health Ontario (PHO), with the most specimens received in 2019 and 2020 from three provinces each.

Of the 169 cyclosporiasis case patients, duplicate and triplicate specimens were received from 12 different patients from PHO. Triplicate specimens were received for one patient in 2014, duplicate specimens were received from two patients and triplicate specimens were received from two patients in 2017, duplicate specimens were received from two patients and triplicate specimens were received from three patients in 2019, and duplicate specimens were received from two patients in 2020.

### 3.2. Genotyping Success

In total, 97.9% (183/187) of the specimens had sequencing data associated with part of one of the markers in the TADS assay, with 11.2% (21/187) of the specimens having at least partial sequencing data for all eight markers. On average, the specimens had sequencing data associated with five of the eight markers.

The most sensitive nuclear markers in the TADS subtyping tool were Nu_378 and Nu_360i2. Sequencing data were obtained for 96.3% (180/187) of the specimens with the Nu_378 marker and 92.5% (173/187) with the Nu_360i2 marker. The Nu_CDS1 marker was the least sensitive nuclear marker, with only 39.0% (73/187) of the specimens generating sequencing data. The mitochondrial marker with the highest sequencing success rate was Mt_MSR with 97.3% (182/187) of the specimens having data for at least one part of the marker, while Mt_Cmt had only 39.0% (73/187) of the specimens with sequencing data (Table 2).

#### 3.2.1. Nuclear Markers

To assess the distribution of haplotypes found within Canadian cyclosporiasis cases for the eight markers, a representative for each case patient was chosen based on the highest sequencing read depth.

**Figure 2:**
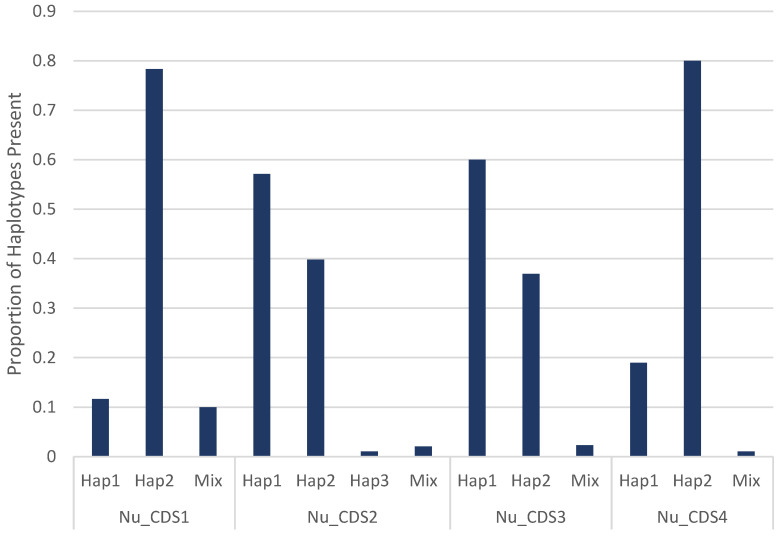
Distribution of haplotypes observed for the Nu_CDS1-4 markers of Canadian *Cyclospora cayetanensis*-positive fecal specimens from 2010 to 2021. The majority of these specimens were homozygous at these four loci, with ≤10% of the specimens being heterozygous at either Part A or Part B of the loci or both. A new haplotype (haplotype 3: Hap2 in Part A and Hap3 in Part B) was recorded at the Nu_CDS2 loci in a 2010 specimen.

Two haplotypes were recorded for all Nu_CDS1-4 markers, except at the Nu_CDS2 locus. A novel Nu_CDS2 haplotype (haplotype 3) was observed in a 2010 specimen that has yet to be described in the CDC’s reference database. For the Nu_CDS1 and Nu_CDS4 markers, haplotype 2 was the most dominant allele observed, being reported in 78.3% (47/60) and 80.0% (76/95) of cyclosporiasis cases, respectively. Conversely, a more even distribution of the two haplotypes was observed for the Nu_CDS2 and Nu_CDS3 markers, with haplotype 1 being detected in 57.1% (56/98) and 60.0% (78/130) of cyclosporiasis cases, respectively. Mixed haplotypes (Hap1/Hap2) at Part A and/or Part B of the Nu_CDS1-4 markers accounted for 10.0% (6/60), 2.0% (2/98), 2.3% (3/130), and 1.1% (1/95) of the specimens with sequencing data at the respective markers (Figure 2).

The Nu_378 and Nu_360i2 markers were more discriminatory than the four Nu_CDS markers. In the Nu_378 marker, the ends of the marker showed the greatest diversity. Five haplotypes were observed in Part A, two haplotypes were observed in Part B, three haplotypes were observed in Part C, and nine haplotypes were observed in Part D (Table 3). Of the 140 cyclosporiasis cases with at least partial haplotypes observed, 90.7% (127) had heterozygous haplotypes described at one or more parts of the marker.

In the Nu_360i2 marker, five haplotypes were observed in Part A, two haplotypes in Part B, four haplotypes in Part C, three haplotypes in Part D, five haplotypes in Part E, and two haplotypes in Part F (Table 4). Heterozygous haplotypes observed in at least one of the marker parts were observed in 89.4% (118) of the 132 cyclosporiasis cases that had at least partial haplotypes observed. The ends of this marker are not consistently more diverse, unlike the Nu_378 marker.

#### 3.2.2. Mitochondrial Markers

Of the 164 cyclosporiasis cases with at least partial haplotypes described for the Mt_MSR marker, 97.6% (160) reported single haplotypes at the parts described. Of the six parts, four haplotypes (Hap1, Hap2, Hap3, and Hap4) were observed in Part A; one haplotype (Hap1) was observed in Parts B, C, and E; and two haplotypes (Hap1 and Hap2) were observed in both Parts D and F. In total, 116 of the cyclosporiasis cases had single haplotypes reported for all six parts, of which five different combinations were observed (Table 5).

In specimens tested prior to 2021, the success rate of defining a haplotype at the mitochondrial junction region (Mt_Cmt) was 34.5% (50/145). However, after changing the primers in 2021 to target only the junction region, the sequencing success rate for this marker improved to 91.7% (22/24).

Specimens collected in 2020 or earlier were Sanger sequenced to gain insight into the haplotypes found in Canada. Combining the Sanger and TADS data for all years, haplotype data for the Mt_Cmt marker were collected for 88.2% (149/169) of cyclosporiasis cases. In total, 10 haplotypes were observed (Table 6). The most abundant haplotype was Cmt154.A (Hap3) representing 51.0% (76/149) of cases with sequencing data at this marker, followed by Cmt154.B (Hap4) in 18.8% (28/149) of cases. Mixed haplotypes were observed in one specimen. One novel haplotype was discovered based on the Sanger sequencing data of a 2012 specimen and was named Cmt154.F to follow the standard nomenclature outlined in Nascimento et al. [18].

### 3.3. Genetic Clustering

**Figure 3:**
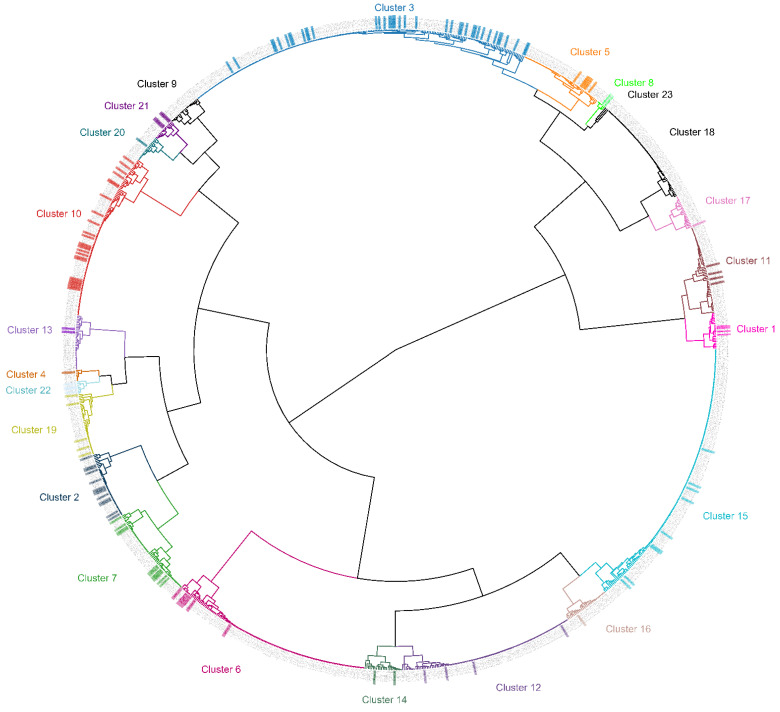
Cluster dendrogram of Canadian *Cyclospora cayetanensis* positive fecal specimens from 2010 to 2021. The dendrogram was generated by applying Ward’s clustering method to the ensemble matrix of pairwise distances that was calculated from 137 Canadian cyclosporiasis case patients (colored) that passed the sequencing requirements and 1077 US specimens from 2018 and 2019 (gray) that were provided with the software as a reference population. Twenty genetic clusters were identified by Module 3, which contained Canadian cyclosporiasis cases (colored), and the number of specimens pertaining to each cluster are as follows: cluster 1 (*n* = 3), cluster 2 (*n* = 12), cluster 3 (*n* = 38), cluster 4 (*n* = 1), cluster 5 (*n* = 5), cluster 6 (*n* = 8), cluster 7 (*n* = 11), cluster 8 (*n* = 2), cluster 10 (*n* = 22), cluster 11 (*n* = 5), cluster 12 (*n* = 4), cluster 13 (*n* = 2), cluster 14 (*n* = 2), cluster 15 (*n* = 7), cluster 16 (*n* = 2), cluster 17 (*n* = 1), cluster 19 (*n* = 4), cluster 20 (*n* = 1), cluster 21 (*n* = 4), and cluster 22 (*n* = 3).

The ensemble-based pairwise distance matrix calculated from the Calculation of a Distance Matrix module (Module 2) of the CDC’s typing workflow [19,21,22], including both Canadian and US reference specimens from 2018 and 2019, is presented as a cluster dendrogram in Figure 3. Of the 169 Canadian cyclosporiasis cases, 81.1% (137) had adequate sequencing information (four or five markers out of the eight markers in the TADS assay, depending on the markers sequenced [21]) to be successfully clustered. The Genetic Cluster Delineation module (Module 3) of the CDC’s typing workflow [19,21,22] predicted that the Canadian cyclosporiasis cases grouped into 20 genetic clusters. There were multiple genetic clusters associated with each year where multiple cases were analyzed (Table 7).

To further evaluate the ensemble-based method in calculating pairwise distances, we clustered repeat specimens from the same cyclosporiasis case patients. In total, duplicate specimens from 10 cyclosporiasis case patients had sufficient data to be clustered. Of the 10 case patients, 9 cases had both duplicate specimens cluster to the same group. In one case, two specimens obtained from the same case patient clustered into two separate groups. One of the duplicate specimens (Specimen A) had six genetic markers fully defined (Nu_CDS2, Nu_CDS3, Nu_CDS4, Nu_378, Nu_360i2, and Mt_MSR). The other specimen (Specimen B) had five genetic markers fully defined (Nu_CDS2, Nu_CDS3, Nu_CDS4, Nu_378, and Mt_MSR) and two markers only partially defined (Nu_CDS1 (Part B) and Nu_360i2 (Part A–E)). Of the fully defined markers in common, the genotypes were the same with the exception that an extra haplotype was defined in Part A (Hap5) and Part B (Hap2) in the Nu_378 marker.

Moreover, we observed changes in the genetic cluster assignment on specimens that underwent sequencing twice. For instance, a 2021 ON specimen was initially fully genotyped at the two mitochondrial markers (Mt_MSR and Mt_Cmt), and partial genotyping data were collected at the Nu_378 and Nu_360i2 markers. Although the specimen passed the clustering requirements, it was resequenced to then gain full genotyping data at all markers, except for Nu_360i2, where a partial genotype was observed, and no genotyping data were obtained for the Nu_CDS1 marker. Similar haplotypes were observed in both sequencing runs of the same specimens, except for Part C in the Nu_378 and Nu_360i2 markers. Hap1 and Hap2 were reported in Part C in the Nu_378 marker in the first round of sequencing, and only Hap2 was reported in the second round. Hap2 was reported in Part C of the Nu_360i2 marker in the first round of sequencing, and Hap1 and Hap2 were reported in the second sequencing run. When clustering the initial data, the specimen clustered in Cluster 20; however, with the additional genotyping data, the specimen then clustered in Cluster 6 using the same reference database. Therefore, clustering is dependent on the number of markers with genotyping data.

## 4. Discussion

We successfully applied the CDC’s TADS genotyping method for *C. cayetanensis* as described by Nascimento et al. [21] to Canadian cyclosporiasis cases and analyzed the data using the bioinformatics pipeline developed by Barratt et al. [22]. Genotyping data were gathered for at least one marker for 97.9% (183/187) of the Canadian *C. cayetanensis*-positive fecal specimens collected from 2010 to 2021. Of the cyclosporiasis case patients, 81.1% (137/169) met the minimum requirements to genetically cluster to other related specimens. This compares to the 78.8% (875/1110) genotyping success rate reported by Barratt et al. [22] when examining their 2019 US specimens using the same data analysis method.

Of the six nuclear markers in the scheme, the four Nu_CDS markers had the fewest haplotypes reported. The Nu_CDS1 marker was the least sensitive with sequencing data (Part A and/or Part B) generated from only 39.0% of the specimens. This is lower than the 61% sequencing success rate reported by Houghton et al. [20] using Sanger sequencing. The Nu_CDS2 and Nu_CDS4 markers displayed similar sequencing success rates of 60.4% and 61.5%, respectively, while the Nu_CDS3 marker displayed the highest sequencing success rate of the four CDS markers at 77.0%. Similar to what was reported in the USA [20,21], two haplotypes (Hap1 and Hap2) were dominant for all four CDS markers. A new haplotype, haplotype 3, for the Nu_CDS2 marker was discovered in the single 2012 Canadian specimen.

The Nu_378 and Nu_360i2 loci had the highest genotyping success rates, 96.3% and 92.5%, respectively, of the nuclear markers. However, partial haplotypes were reported for 18.2% and 51.3% of the specimens for the Nu_378 and Nu_360i2 markers, respectively, as haplotypes were commonly missing on either the 3′ or 5′ end of the markers. This is likely the result of the tagmentation step during the library preparation favoring higher coverage in the middle of an amplicon than at the ends [21]. In this study, 90.7% and 89.4% of the specimens displayed heterozygous regions in the Nu_378 and Nu_360i2 markers, respectively. This compares to the 92% heterozygous US specimens at the Nu_378 loci and 80% heterozygous specimens at the Nu_360i2 specimens reported by Barratt et al. [19]. With the high genotyping success rates and diverse heterozygous haplotypes present, these two nuclear markers provide good discriminatory power for clustering cyclosporiasis cases.

Similarly to the Nu_378 and Nu_360i2 loci, the Mt_MSR marker displayed a high genotyping success rate of 97.3%. The 24.1% of the specimens with partial haplotypes were missing data on either ends of the marker, similar to the Nu_378 and Nu_360i2 markers. The genotyping success rate for the Mt_Cmt marker was low using the Haplotype Caller module (Module 1) from the CDC *Cyclospora* typing pipeline when using the MT5732F and MT6266R primer set producing a ~534 bp amplicon, but significantly improved when targeting the junction region alone using the 100F and 54R primer set that produces a 109–214 bp amplicon. One possible explanation is that the repeat region has less coverage since it is on the end of the larger amplicon, making it challenging to accurately classify the repeat region. Sanger sequencing data were also collected for this region. In total, 10 haplotypes were discovered in the Canadian specimens, with a distribution comparable to those described in US studies [18]. Cmt154.A is the most dominant allele in our specimens, followed by Cmt154.B, as in Nascimento et al. [18]. Homozygous haplotypes were described for 97.5% of the cases at the Mt_MSR locus and 99.3% of the cases at the Mt_Cmt locus.

In total, 20 genetic clusters were observed in the Canadian fecal specimens tested. We observed that, in the years with higher numbers of specimens genotyped (2019 to 2021), multiple genetic clusters were identified. This indicates that there are likely multiple sources of infection in a given outbreak season. It is also observed that multiple clusters persist over several years. For instance, four clusters (Clusters 2, 3, 7, and 10) contained at least two isolates from 2019, 2020, and 2021. Our genotyping results suggest that the TADS scheme has potential for real-time case-linkage investigations. Of our samples, 81.1% had sufficient genetic data to cluster. With this success rate during real-time outbreak investigations, the TADS scheme would be a key tool for linking cases together with epidemiological data, especially when outbreaks are caused by multiple sources of infection. More meaningful conclusions about the clustering results require a further comparative study against the epidemiological data to verify that the clusters are epidemiologically linked.

In the case of one of the 2021 ON specimens, its cluster assignment changed when the specimen was resequenced to improve the genotyping data. This observation highlights that when samples just meet the minimum requirements to be genetically clustered, they may not be clustered reliably. This may indicate that the minimum number of sequenced markers currently required to cluster specimens (four or five out of eight, depending on the markers sequenced [21]) should be increased to at least five or six out of the eight markers. This observation also suggests that the method would benefit from sequencing additional markers to improve clustering reliability. With the low sequencing success rate of the Nu_CDS1, Nu_CDS2, and Nu_CDS4 markers, specimens are more likely to have missing data than if additional reliable markers with performance comparable to the Nu_378 and Nu_360i2 markers were available. Furthermore, the clustering of specimens is dependent on both the size of the reference database and the number of specimens sequenced [19]. The data analysis modules more accurately cluster linked specimens together when there is a larger dataset, making it imperative to genotype as many specimens as possible during outbreak seasons.

There were some challenges associated with this study. For instance, the specimens genetically analyzed in this study represented a limited subset (from 2% to 32% of cases reported) of the already small number of laboratory-confirmed cyclosporiasis cases across Canada. This is because the majority of *Cyclospora*-positive specimens in Canada are stored in SAF (sodium acetate–acetic acid–formalin) to preserve the parasite for microscopic examination. Unfortunately, the formalin cross-links and denatures the DNA [23], hindering downstream molecular analysis. In addition, the unpreserved specimens were collected from laboratories that use multiplex qPCR assays to diagnose parasitic infections. These specimens originated from specific regions in only 4 provinces (ON, QC, BC, and NL) of the 10 in Canada, introducing bias in the genotypes observed because they only represent a subset of geographic areas within Canada.

Additional difficulties are inherent in working with *C. cayetanensis* because it cannot be propagated in the lab. Only picogram quantities of DNA can be isolated from clinical specimens for sequencing; consequently, routine WGS genotyping is not yet practical for this parasite. While the TADS scheme [21,22] investigated in the current study is more likely to succeed than WGS, it only considers eight small regions in the nuclear and mitochondrial genomes, so it is important to find highly descriptive markers. Since *C. cayetanensis* is sexually reproducing, heterozygous markers provide greater genetic insights into the infecting isolate. Therefore, if further sensitive and discriminatory markers are found for this parasite, combined with the ensemble-based distance statistic tool [19,21,22] to handle heterozygous and complex genotyping data, the TADS scheme should provide useful insights into clustering cyclosporiasis cases together with epidemiological case-linkage data to mitigate outbreaks.

## 5. Conclusions

This is the first study to successfully genetically analyze Canadian clinical cyclosporiasis infections. With the CDC’s *C. cayetanensis* TADS genotyping scheme comprising eight markers, we were able to obtain genetic information for at least one marker in 97.9% of the specimens. We found that the majority of Canadian *C. cayetanensis* genotypes were identical to those described in the United States [20,21].

With these genetic data, 20 clusters were identified from clinical samples spanning a 10-year period. We observed that there were multiple genetic clusters for the last 3 years, suggesting multiple sources of infection during the multijurisdictional outbreak seasons that were declared in Canada. The TADS scheme enabled us to associate cyclosporiasis cases to one another genetically in this study. The next step would be to compare the epidemiological data of the cyclosporiasis cases genotyped in this study with the genetic clusters we have identified to assess how successfully this tool is likely to perform during real-time outbreak investigations. The results generated in this study are promising and suggest that the TADS genotyping scheme will be very useful in supporting real-time outbreak investigations because we were able to relate select specimens with similar genetic data to one another.

In order to improve the genotyping ability of the TADS scheme, novel markers that display a greater sequencing success rate and higher entropy should replace the least sensitive and discriminatory markers, Nu_CDS1, Nu_CDS2, and Nu_CDS4. Research should focus on identifying new markers through comparative analyses of whole genome sequences available to date, including the newly published hybrid assembly of a Canadian *C. cayetanensis* isolate [24] and four other Canadian genomes generated (unpublished). Additionally, comparative analyses should focus on finding discriminatory markers that further differentiate the isolates that are grouping into persistent clusters observed over several years. By finding the most sensitive and descriptive markers, we will gain a more complete genetic picture of *C. cayetanensis* isolates, enabling more reliable clustering. These genetic data, combined with epidemiological data, will help to improve Canadian outbreak investigations and to better understand where *C. cayetanensis* infections are originating.

## Figures and Tables

**Table 1:** Modified PCR reaction conditions used for the Cyclospora cayetanensis TADS subtyping scheme ^1^.

Gene Target	Length	Primers (Concentration)	Primer Sequence (5′-3′)	PCR Conditions	
Nu_CDS1	175 bp	GT1-F (600 nM) GT1-R (600 nM)	CTCCTTGCTGCTCAGAACGA CAAGAGAGGAGCAGTGGCAA	Initial denaturation:	98 °C for 2 min
				35 cycles of:	98 °C for 15 s
					67 °C for 30 s
					65 °C for 30 s
				Final extension:	65 °C for 5 min
				Hold:	4 °C
Nu_CDS2	246 bp	GT2-F (200 nM) GT2-R (200 nM)	TGCAAACTACTAAGGGCGCA CGCCTTCTCTTGAGCCTTGA	Initial denaturation: 35 cycles of: Final extension: Hold:	98 °C for 2 min 98 °C for 15 s 67 °C for 15 s 65 °C for 15 s 65 °C for 5 min 4 °C
Nu_CDS3	220 bp	GT3-F (400 nM) GT3-R (400 nM)	AATCGAATCGGTGCAGTGCTTA GACTGAACGTGTGAGAGGGG		
Nu_CDS4	179 bp	GT4-F (400 nM) GT4-R (400 nM)	GTAGATGGGTCCTTGAAGGCT CAGACGCCTAAGGAACCGAA		
Nu_378	469 bp	HC378F (400 nM) HC378R (400 nM)	CCCCTGCCTTGTTCTTGGTGAA CCGGCGACACAGAGGTACC		
Nu_360i2	650 bp	HC360i2F (400 nM) HC360i2R (400 nM)	CCCATTACGCCGCATAGAGT GCATTGCAAAGCCAGTCAGC	Initial denaturation: 35 cycles of: Final extension: Hold:	98 °C for 2 min 98 °C for 15 s 67 °C for 30 s 72 °C for 30 s 72 °C for 5 min 4 °C
Mt_MSR	674 bp	15F (200 nM) 688R (200 nM)	GGACATGCAGTAACCTTTCCG AGGAAAGGTTAACCGCTGTCA	Initial denaturation: 35 cycles of: Final extension: Hold:	94 °C for 2 min 94 °C for 15 s 55 °C for 30 s 68 °C for 40 s 68 °C for 5 min 4 °C
Mt_Cmt	±534 bp 109–214 bp	MT5732F (200 nM) MT6266R (200 nM) 100F (200 nM) ^2^ 54R (200 nM) ^2^	GTCGTTACACCATTCATGCAG CCCAAGCAATCGGATCGTGTT TACCAAAGCATCCATCTACAGC CCCAAGCAATCGGATCGTGTT		

^1^ Developed by Nascimento et al. [21], except for the ±534 bp Mt_Cmt primers that were first published in Barratt et al. [22]. ^2^ Primers changed for 2021 specimens, targeting only the junction region [18,21].

**Table 2:** Sequencing success rate observed for each marker in the TADS assay (*n* = 187).

Marker	Full Haplotype ^1^	Partial Haplotype ^2^	No Data
Nu_CDS1	61 (32.6%)	12 (6.4%)	114 (61.0%)
Nu_CDS2	104 (55.6%)	9 (4.8%)	74 (39.6%)
Nu_CDS3	142 (75.9%)	2 (1.1%)	43 (23.0%)
Nu_CDS4	104 (55.6%)	11 (5.9%)	72 (38.5%)
Nu_378	146 (78.1%)	34 (18.2%)	7 (3.7%)
Nu_360i2	77 (41.2%)	96 (51.3%)	14 (7.5%)
Mt_MSR	137 (73.3%)	45 (24.1%)	5 (2.7%)
Mt_Cmt	73 (39.0%)	Not applicable	114 (61.0%)

^1^ All parts of the marker had sequencing data associated to it. ^2^ At least one part of the marker had sequencing data associated to it.

**Table 3:** Haplotypes present in the four parts of the Nu_378 gene ^1^.

Part A	Part B	Part C	Part D
Hap2	Hap1	Hap1	Hap1
Hap4	Hap2	Hap2	Hap2
Hap5		Hap4	Hap3
Hap6			Hap4
Hap8			Hap5
			Hap6
			Hap7
			Hap8
			Hap11

^1^ Further information of Parts A through D of the Nu_378 marker can be found in the Supplemental File S1.

**Table 4:** Haplotypes present in the six parts of the Nu_360i2 gene ^1^.

Part A	Part B	Part C	Part D	Part E	Part F
Hap1	Hap1	Hap1	Hap1	Hap1	Hap1
Hap2	Hap2	Hap2	Hap2	Hap2	Hap3
Hap3		Hap3	Hap3	Hap3	
Hap4		Hap4		Hap6	
Hap5				Hap8	

^1^ Further information of Parts A through F of the Nu_360i2 marker can be found in the Supplemental File S1.

**Table 5:** Haplotype combinations of the Mt_MSR marker ^1^ in homozygous infections (*n* = 116).

Combination	No. of Cases (%)	Part A	Part B	Part C	Part D	Part E	Part F
1	13 (11.4%)	Hap1	Hap1	Hap1	Hap1	Hap1	Hap1
2	71 (62.3%)	Hap1	Hap1	Hap1	Hap1	Hap1	Hap2
3	1 (0.1%)	Hap1	Hap1	Hap1	Hap2	Hap1	Hap2
4	13 (11.4%)	Hap2	Hap1	Hap1	Hap1	Hap1	Hap1
5	18 (15.7%)	Hap3	Hap1	Hap1	Hap1	Hap1	Hap2

^1^ Further information of Parts A through F of the Mt_MSR marker can be found in the Supplemental File S1.

**Table 6:** Mitochondrial junction region (Mt_Cmt) haplotypes observed in Canadian *Cyclospora cayetanensis*-positive fecal specimens (*n* = 149).

Mt_Cmt Junction Name	Haplotype No.	Number of Cases (%)
Cmt154.A	Hap3	76 (51.0%)
Cmt154.B	Hap4	28 (18.8%)
Cmt169.A	Hap8	15 (10.1%)
Cmt169.B	Hap9	10 (6.7%)
Cmt184.B	Hap12	13 (8.7%)
Cmt184.C	Hap13	1 (0.7%)
Cmt199.A	Hap17	2 (1.3%)
Cmt199.C	Hap19	1 (0.7%)
Cmt214.X	Hap25	1 (0.7%)
Cmt154.F ^1^	Hap39	1 (0.7%)
Mix	N/A	1 (0.7%)

^1^ Novel haplotype found in this study.

**Table 7:** Genetic clusters observed in Canadian cyclosporiasis cases by year.

Year	No. of Specimens Clustered (%)	Total No. of Clusters (*n* ≥ 1)	Clusters Observed (*n* > 1)
2010 (*n* = 1)	1 (100%)	1	
2012 (*n* = 1)	1 (100%)	1	
2014 (*n* = 2)	2 (100%)	2	
2015 (*n* = 10)	9 (90%)	6	Cluster 1 (*n* = 2)
			Cluster 3 (*n* = 2)
			Cluster 7 (*n* = 2)
2016 (*n* = 3)	2 (67%)	2	
2017 (*n* = 13)	9 (69%)	6	Cluster 3 (*n* = 3) Cluster 22 (*n* = 2)
2018 (*n* = 4)	4 (100%)	4	
2019 (*n* = 55)	41 (75%)	18	Cluster 2 (*n* = 2) Cluster 3 (*n* = 9) Cluster 7 (*n* = 2) Cluster 8 (*n* = 2) Cluster 10 (*n* = 3) Cluster 11 (*n* = 4) Cluster 12 (*n* = 3) Cluster 15 (*n* = 4) Cluster 19 (*n* = 2) Cluster 22 (*n* = 2)
2020 (*n* = 56)	37 (66%)	10	Cluster 2 (*n* = 6) Cluster 3 (*n* = 14) Cluster 6 (*n* = 5) Cluster 7 (*n* = 3) Cluster 10 (*n* = 3) Cluster 15 (*n* = 2)
2021 (*n* = 24)	21 (88%)	9	Cluster 2 (*n* = 2) Cluster 3 (*n* = 3) Cluster 5 (*n* = 4) Cluster 6 (*n* = 2) Cluster 7 (*n* = 3) Cluster 10 (*n* = 4)

## Data Availability

Raw sequence reads are available under BioProject no. PRJNA796535.

## Supplementary Materials

The following supporting information can be downloaded at: https://www.mdpi.com/article/10.3390/microorganisms10020447/s1: File S1: *Cyclospora cayetanensis* TADS markers and haplotypes.
